# S2k guideline “Calculated parenteral initial treatment of bacterial infections in adults – update 2018”, 2^nd^ updated version: Foreword

**DOI:** 10.3205/id000064

**Published:** 2020-03-26

**Authors:** Klaus-Friedrich Bodmann, Béatrice Grabein, Michael Kresken

**Affiliations:** 1 Klinik für Internistische Intensiv- und Notfallmedizin und Klinische Infektiologie, Klinikum Barnim GmbH, Werner Forßmann Krankenhaus, Eberswalde, Germany; 2 Stabsstelle Klinische Mikrobiologie und Krankenhaushygiene, Klinikum der Universität München, Munich, Germany; 3 Antiinfectives Intelligence GmbH, Campus Hochschule Bonn-Rhein-Sieg, Rheinbach, Germany; 4 Rheinische Fachhochschule Köln gGmbH, Cologne, Germany

## Foreword

This guideline by the Paul-Ehrlich-Gesellschaft für Chemotherapie e.V. (PEG) is an update of the “Recommendations on calculated parenteral initial therapy of bacterial diseases in adults” from 2010 [1], [2], [3]. 

The 2^nd^ version of the guideline “Calculated parenteral initial treatment of bacterial infections in adults – update 2018“ in German was created on July 25 2019 [4]. It has been translated to address an international audience.

The responsibility for the content of each chapter lies with the respective authors.

The guideline is divided into 17 chapters, which are published here as 17 single articles:

1. Introduction and antibiotics [5]
2. Microbiology [6]
3. Pharmacokinetics and pharmacodynamics [7]
4. Safety and tolerability [8]
5. Respiratory infections [9]
6. Infections in the ear, nose, throat and mouth and jaw area [10]
7. Intra-abdominal infections [11]
8. Infections of the kidneys and the genito-urinary tract [12]
9. Skin and soft tissue infections [13]
10. Bone and joint infections [14]
11. Sepsis [15]
12. Bacterial endocarditis [16]
13. Bacterial meningitis [17]
14. Bacterial gastrointestinal infections [18]
15. Antibiotic treatment in the elderly [19]
16. Infections with multi-resistant Gram-negative rods – ESBL producers, carbapenemase-producing Enterobacteriaceae, carbapenem-resistant *Acinetobacter baumannii *[20]
17. Economic aspects of antibiotic treatment [21]

Following the publication of the 1^st^ version of the guideline in German, a few dosage suggestions were updated by the working groups. The exact changes are given in the single articles. 

## Authors and contributing societies

With the participation of:

- Berufsverband Deutscher Anästhesisten e.V. (BDA)
- Deutsche Dermatologische Gesellschaft e.V. (DDG)
- Deutsche Gesellschaft für Allgemein- und Viszeralchirurgie e.V. (DGAV)
- Deutsche Gesellschaft für Anästhesiologie und Intensivmedizin e.V. (DGAI)
- Deutsche Gesellschaft für Geriatrie e.V. (DGG)
- Deutsche Gesellschaft für Hygiene und Mikrobiologie e.V. (DGHM)
- Deutsche Gesellschaft für Hals-Nasen-Ohren-Heilkunde, Kopf- und Hals-Chirurgie e.V. (DGHNOKHC)
- Deutsche Gesellschaft für Infektiologie e.V. (DGI)
- Deutsche Gesellschaft für Innere Medizin e.V. (DGIM)
- Deutsche Gesellschaft für Krankenhaushygiene e.V. (DGKH)
- Deutsche Gesellschaft für Mund-, Kiefer- und Gesichtschirurgie e.V. (DGMKG)
- Deutsche Gesellschaft für Orthopädie und Unfallchirurgie e.V. (DGOU)
- Deutsche Gesellschaft für Pneumologie e.V. (DGP)
- Deutsche Gesellschaft für Urologie e.V. (DGU)
- Österreichische Gesellschaft für Antimikrobielle Chemotherapie (ÖGACH)
- Österreichische Gesellschaft für Hygiene, Mikrobiologie und Präventivmedizin (ÖGHMP)
- Österreichische Gesellschaft für Infektionskrankheiten und Tropenmedizin (ÖGIT)

Authors:

- Klaus-Friedrich Bodmann^1^
- Béatrice Grabein^2^
- Michael Kresken^3,4^
- Hartmut Derendorf^5^
- Ralf Stahlmann^6^
- Sebastian R. Ott^7^
- Bernhard Olzowy^8^
- Christian Eckmann^9^
- Florian Wagenlehner^10^
- Cord Sunderkötter^11^
- Mathias G. Vossen^12^
- Pascal M. Dohmen^13^
- Pramod M. Shah^14^
- Reinier Mutters^15^
- Peter Walger^16^
- Michael Wilke^17 ^

for the expert panel*

^1^ Klinik für Internistische Intensiv- und Notfallmedizin und Klinische Infektiologie, Klinikum Barnim GmbH, Werner Forßmann Krankenhaus, Eberswalde, Germany

^2^ Stabsstelle Klinische Mikrobiologie und Krankenhaushygiene, Klinikum der Universität München, Munich, Germany

^3^ Antiinfectives Intelligence GmbH, Campus Hochschule Bonn-Rhein-Sieg, Rheinbach, Germany

^4^ Rheinische Fachhochschule Köln gGmbH, Cologne, Germany

^5^ Department of Pharmaceutics, College of Pharmacy, University of Florida, Gainesville, USA

^6^ Institut für Klinische Pharmakologie und Toxikologie, Charité – Universtitätsmedizin Berlin, Berlin, Germany

^7^ Universitätsklinik für Pneumologie, Universitätsspital Bern (Inselspital) und Universität Bern, Bern, Switzerland

^8^ HNO-Zentrum Landsberg am Lech, Landsberg am Lech, Germany

^9^ Klinik für Allgemein-, Viszeral- und Thoraxchirurgie, Klinikum Peine, Peine, Germany

^10^ Justus Liebig Universität Gießen, Klinik und Poliklinik für Urologie, Kinderurologie und Andrologie, Gießen, Germany

^11^ Universitätsklinik und Poliklinik für Dermatologie und Venerologie, Martin-Luther-Universität Halle-Wittenberg, Halle (Saale), Germany

^12^ Medizinische Universität Wien, Universitätsklinik für Innere Medizin I, Klinische Abteilung für Infektionen & Tropenmedizin, Allgemeines Krankenhaus Wien, Vienna, Austria

^13^ Klinik und Poliklinik für Herzchirurgie, Universitätsmedizin Rostock, Rostock, Germany

^14^ Auf dem Mühlberg 30c, 60599 Frankfurt am Main, Germany

^15^ Institut für Medizinische Mikrobiologie und Krankenhaushygiene, Philipps-Universität Marburg, Marburg, Germany

^16^ Verbund Katholischer Kliniken Düsseldorf, Zentralbereich Hygiene, Infektionsmanagement und ABS, Düsseldorf, Germany

^17^ inspiring-health Dr. Wilke GmbH, Munich, Germany

* Other members of the expert group: Bilal Al-Nawas, Mainz; Karsten Becker, Münster; Alexander Brinkmann, Heidenheim; Reinhard Brodt, Frankfurt am Main; Michael Ebenhoch, Murnau; Reinhard Fünfstück, Weimar; Rainer Gattringer, Linz, Österreich; Wolfgang Graninger, Vienna, Austria; Miriam Havel, Munich; Tobias Heinrichs, Gainesville, Florida, USA; Hans Jürgen Heppner, Schwelm; Gunnar Hischebeth, Bonn; Gert Höffken, Dresden; Rainer Höhl, Nürnberg; Udo Hoyme, Arnstadt; Claudia Hübner, Greifswald; Rainer Isenmann, Ellwangen; Wolfgang Kämmerer, Augsburg; Julia Karbach, Mainz; Martin Kolditz, Dresden; Wolfgang Krüger, Konstanz; Ernst Kühnen, Trier; Peter Kujath, Lübeck; Cordula Lebert, Nürnberg; Hartmut Lode, Berlin; Christoph Lübbert, Leipzig; Matthias Militz, Murnau; Rainer Müller, Dresden; Kurt Naber, Straubing; Roland Nau, Göttingen; Adrian Pilatz, Gießen; Mathias W. Pletz, Jena; Annette Pross, Regensburg; Tobias Reimers, Gainesville, Florida, USA; Arne C. Rodloff, Leipzig; Franz-Josef Schmitz, Minden; Helmut Schöfer, Frankfurt/Main; Sören Schubert, München; Eberhard Straube, Jena; Florian Thalhammer, Wien, Österreich; Thomas A. Wichelhaus, Frankfurt am Main; Birgit Willinger, Vienna, Austria.

Representatives of the participating professional bodies:

- Bilal Al-Nawas, Mainz, Germany (Deutsche Gesellschaft für Mund-, Kiefer- und Gesichtschirurgie e.V., DGMKG)
- Klaus-Friedrich Bodmann, Eberswalde, Germany (Deutsche Gesellschaft für Innere Medizin e.V., DGIM)
- Alexander Brinkmann, Heidenheim, Germany (Berufsverband Deutscher Anästhesisten e.V., BDA; Deutsche Gesellschaft für Anästhesiologie und Intensivmedizin e.V., DGAI)
- Christian Eckmann, Peine, Germany (Deutsche Gesellschaft für Allgemein- und Viszeralchirurgie e.V., DGAV)
- Béatrice Grabein, Munich, Germany (Deutsche Gesellschaft für Hygiene und Mikrobiologie e.V., DGHM)
- Hans Jürgen Heppner, Schwelm, Germany (Deutsche Gesellschaft für Geriatrie e.V., DGG)
- Caroline Isner, Berlin, Germany (Deutsche Gesellschaft für Infektiologie e.V., DGI)
- Julia Karbach, Mainz, Germany (Deutsche Gesellschaft für Mund-, Kiefer- und Gesichtschirurgie e.V., DGMKG)
- Martin Kolditz, Dresden, Germany (Deutsche Gesellschaft für Pneumologie e.V., DGP)
- Robert Krause, Graz, Austria (Österreichische Gesellschaft für Antimikrobielle Chemotherapie, ÖGACH)
- Wolfgang Krüger, Konstanz, Germany (Berufsverband Deutscher Anästhesisten e.V., BDA; Deutsche Gesellschaft für Anästhesiologie und Intensivmedizin e.V., DGAI)
- Matthias Militz, Murnau, Germany (Deutsche Gesellschaft für Orthopädie und Unfallchirurgie e.V., DGOU)
- Bernhard Olzowy, Landsberg, Germany (Deutsche Gesellschaft für Hals-Nasen-Ohren-Heilkunde, Kopf- und Hals-Chirurgie e.V., DGHNOKHC)
- Cord Sunderkötter, Halle/Saale, Germany (Deutsche Dermatologische Gesellschaft e.V., DDG)
- Florian Thalhammer, Vienna, Austria (Deutsche Gesellschaft für Innere Medizin e.V., DGIM; Österreichische Gesellschaft für Infektionskrankheiten und Tropenmedizin, ÖGIT)
- Florian Wagenlehner, Gießen, Germany (Deutsche Gesellschaft für Urologie e.V., DGU)
- Peter Walger, Bonn, Germany (Deutsche Gesellschaft für Krankenhaushygiene e.V., DGKH)
- Birgit Willinger, Vienna, Austria (Österreichische Gesellschaft für Hygiene, Mikrobiologie und Präventivmedizin, ÖGHMP)

## Notes

### Competing interests

The authors declare that they have no competing interests.
